# Effects of Long-term Diving Training on Cortical Gyrification

**DOI:** 10.1038/srep28243

**Published:** 2016-06-20

**Authors:** Yuanchao Zhang, Lu Zhao, Wenwei Bi, Yue Wang, Gaoxia Wei, Alan Evans, Tianzi Jiang

**Affiliations:** 1 Key Laboratory for NeuroInformation of Ministry of Education, School of Life Science and Technology, University of Electronic Science and Technology of China, Chengdu 610054, China; 2 Montreal Neurological Institute, McGill University, Montreal, Quebec, H3A2B4, Canada; 3 Key Laboratory of Behavioral Science, Institute of Psychology, Chinese Academy of Sciences, 100101 Beijing, China

## Abstract

During human brain development, cortical gyrification, which is believed to facilitate compact wiring of neural circuits, has been shown to follow an inverted U-shaped curve, coinciding with the two-stage neurodevelopmental process of initial synaptic overproduction with subsequent pruning. This trajectory allows postnatal experiences to refine the wiring, which may manifest as endophenotypic changes in cortical gyrification. Diving experts, typical elite athletes who commence intensive motor training at a very young age in their early childhood, serve ideal models for examining the gyrification changes related to long-term intensive diving training. Using local gyrification index (LGI), we compared the cortical gyrification between 12 diving experts and 12 controls. Compared with controls, diving experts showed widespread LGI reductions in regions relevant to diving performance. Negative correlations between LGIs and years of diving training were also observed in diving experts. Further exploratory network efficiency analysis of structural cortical networks, inferred from interregional correlation of LGIs, revealed comparable global and local efficiency in diving experts relative to controls. These findings suggest that gyrification reductions in diving experts may be the result of long-term diving training which could refine the neural circuitry (via synaptic pruning) and might be the anatomical substrate underlying their extraordinary diving performance.

The human cerebral cortex is a highly complex structure with rich gyri and sulci. Gyrification, the process of gyrus and sulcus formation, allows a larger cortical surface area (therefore a greater number of neurons) to fit in the skull, facilitating the development of compact wiring for efficient information processing^1^. The exact mechanisms underlying the developmental process of cortical gyrification remain unclear, although a number of theories have surfaced. Among these theories, the most attractive model for the development of cortical gyrification is the tension-based hypothesis proposing that cortical gyrification is mainly driven by the tension of the underlying white matter fiber tracts^2^. Other hypotheses emphasize the developmental mechanisms of the cerebral cortex^3^ or differential expansion of cortical layers^4^ as the major causes for cortical gyrification (the gray matter hypothesis). Recent studies supported the opinion that the tension-based hypothesis and the gray matter hypothesis should be considered to be complementary or coexistent in the causal mechanisms of cortical gyrification, rather than as alternatives^5^.

Cortical gyrification of the human brain begins between 10 and 15 weeks of fetal life^6^,^7^. During the first trimester of fetal life, cortical gyrification increases dramatically^6^, which transforms the cerebral cortical landscape from a relatively smooth, lissencephalic structure into a highly convoluted cortex, resembling the morphology of the adult brain. Existing morphometric studies on the developmental trajectory of human cortical gyrification were mainly conducted using gyrification index (GI) or local gyrification index (LGI), with a higher GI or LGI value indicating a more complex cortical sheet^8^,^9^. The findings of these studies suggest that the GI or LGI continues to increase after birth until it reaches its peak somewhere between 2 and 6 postnatal years^10^,^11^,^12^, and is then followed by a protracted declining period^12^,^13^,^14^. These changes likely reflect the two-stage neurodevelopmental process of initial synaptic overproduction followed by experience-dependent synaptic elimination which represents a fine-tuning process of neural circuitry and plays important roles in neural circuit maturation^1^,^14^,^15^. Morphological studies on twins demonstrated that the cerebral volume is highly determined by genetic variations whereas the cortical gyrification is primarily determined by lifelong experience- or environment-related factors^16^,^17^,^18^. Indeed, recent studies of brain morphometry in musicians showed altered morphometric characteristics in the central sulcus in musicians^19^. Another study documented both increased and decreased cortical gyrification in meditation practitioners compared with controls^20^. The effect of long-term motor training on the morphology of cortical gyrification, however, remains unknown.

Diving is a competitive sport that greatly exceeds those of simple physical movement or locomotion and demands precise motor control, timing, coordination and excellent proprioceptive sensation during the execution of the motions. Diving experts are typical elite athletes that usually commence intensive motor training at a very young age in their early childhood, and therefore serve ideal models for examining the cerebral neuroplastic changes related to long-term intensive diving training. Of note, our previous studies found that diving experts had significant changes in gray matter volume and cortical thickness in multiple brain areas^21^,^22^ as well as regional inflation in the thalamus and pallidum^23^, suggesting possible changes in the intracortical organization and thalamocortical connections^18^. According to the aforementioned tension-based hypothesis and gray matter hypothesis of cortical gyrification^2^,^4^,^5^, alterations in cortical gyrification are also expected in the diving experts.

Using surface-based LGI, the present study aims to investigate cortical gyrification changes in diving experts compared with matched controls. Specifically, we fitted vertex-wise linear model to compare the LGI maps between 12 professional diving experts and 12 controls. We hypothesized that diving experts would show LGI changes in multiple brain regions in response to long-term intensive diving training, including some motor cortices and the parahippocampal cortex. Given structural covariance of LGI has been used to construct structural brain networks^24^, we further performed exploratory network efficiency analyses of the structural cortical networks inferred from interregional correlation of LGIs to examine whether diving experts are more efficient in information processing.

## Materials and Methods

### Subjects

All subjects of this study participated in our previous studies^22^,^23^. The twelve professional diving experts are described in detail in Table 1. In brief, all the twelve diving experts (6 females and 6 males) are national-level masters (mean age, 14.58; SD, 1.68) with top-level diving skills (In China, professional athletes are classified into A, B, C, and D categories to distinguish their competence level, which respectively corresponds to international-level masters, national-level masters, the first-class level and the second-class level). The control group (6 females and 6 males) was matched for age, gender and educational level, and included twelve healthy subjects (mean age, 14.92; SD, 1.38) who were not involved in any extensive physical training or professional experience. All of the subjects were right-handed and were medically and neurologically stable. No subjects had any lifetime histories of substance dependence. Written informed consent from their parents was obtained, and the study was approved by the Institutional Review Board of Beijing MRI Center for Brain Research. Experiments were carried out in accordance with the approved guidelines.

### MRI Data Acquisition

High-resolution anatomical images of the whole brain were acquired on a 3-tesla Trio system (Siemens, Erlangen, Germany) with 12-channel head matrix coil using a magnetisation-prepared rapid-acquisition gradient echo sequence. The following parameters were used for the volumetric acquisition: repetition time = 2530 ms, echo time = 3.37 ms, flip angle = 7 degrees, slice thickness = 1.33 mm, FOV = 256 mm, 512 × 512- pixel matrix. The voxel size was 0.5 × 0.5 × 1.33 mm^3^. The scan time for the T1-weighted sequence was 486 s. During the scanning, each subject reclined in a supine position on the bed of the scanner and was asked to lie still during the imaging time. A foam head holder and padding were placed around the subject’s head. In addition, headphones were provided to block background noise.

### Local Gyrification Index

Each scan was processed using *FreeSurfer* ( http://surfer.nmr.mgh.harvard.edu/) to obtain the LGI. Briefly, the LGI map can be obtained in four steps^8^. First, the pial surface is reconstructed in three-dimensional space. Second, an outer surface can be obtained from the outer hull which tightly wraps the pial surface. Third, the LGI was calculated for each vertex on the outer surface, as a ratio of areas of circular region centered on this vertex and the area of the corresponding region on the pial surface. Hence, LGI is able to quantify the amount of cortical surface invaginated in the sulci and measure the spatial frequency of cortical gyrification and the depth of the sulci. Fourth, the LGI map is obtained by propagating LGI values from the outer surface to the pial surface. For comparison, all of the individual reconstructed cortical surfaces were aligned to an average template by using a surface-based registration algorithm^25^. Then the LGI maps were resampled and smoothed with a heat kernel of 10 mm width.

### Construction of Gyrification-based Networks

Firstly, each individual LGI map was parcellated using the built-in Desikan atlas of *FreeSurfer*, which includes 68 anatomical regions (34 in each hemisphere while excluding the corpus callosum). The LGI for each cortical region was calculated as the average LGI of all vertices in that region. Secondly, the interregional correlation matrix *C* = [*c*_*ij*_] (*i, j* = *1, 2,… N*, here *N* = 68) of each group was obtained by calculating the Pearson correlation coefficients across individuals between the LGIs of every pair of regions^24^. Prior to the correlation analysis, a linear regression was performed at every cortical region to remove the effects of age, gender and intracranial volume (ICV). Finally, the correlation matrix of each group was thresholded into a binarized matrix *B* = [*b*_*ij*_], where *b*_*ij*_ is 1 if the absolute value of the correlation coefficient *c*_*ij*_ between regions *i* and *j* is larger than a given correlation threshold, and 0 otherwise.

### Network Efficiency Measurements

The undirected network (graph) *G* was represented by a binarized matrix *B* with *N* nodes and *K* edges, where nodes are cortical regions and edges indicate undirected links corresponding to its nonzero elements. To ensure the networks of two groups have the same number of edges and are comparable, the correlation matrix *C* of each group was thresholded into a binarized matrix with a fixed sparsity *S*^26^, which is defined as the number of edges *K* in a graph divided by the maximum possible number of edges *N*(*N* − *1*)*/2*. Here, a wide range of sparsity thresholds 10% ≤ *S* ≤ 40% were used.

In this study, we employed the network efficiency measurements to examine the global topological properties of the structural cortical networks obtained for the two groups. For a graph *G* with *N* nodes and *K* edges, the global efficiency *E*_*glob*_ (*G*) is defined as^27^


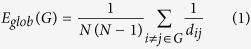


where *d*_*ij*_ is the shortest path length between node *i* and node *j* in *G*. The local efficiency *E*_*loc*_(*G*) is measured as^27^


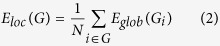


where *G*_*i*_ denotes the subgraph composed of the nearest neighbors of node *i*.

### Statistical Analysis

Vertex-by-vertex contrasts of LGI were performed between diving experts and controls. Specifically, each contrast was entered into a vertex-by-vertex GLM including diagnosis, sex, exact age and ICV as covariates. Subsequently, random field theory (RFT) was used to assess the significance of the statistical maps and to correct for multiple comparisons^28^. Clusters were first reported reaching a significance level of P < 0.05 RFT corrected. Those reaching a looser significance level of P < 0.005 uncorrected were also indicated.

Within the diving experts, vertex-wise correlation analyses were performed to determine the associations between LGI and years of diving training, while removing the partial effect of age, sex and ICV. Then, the same procedures as the above between-group analysis on LGI were repeated to correct for multiple comparisons and to report the results of the correlation analysis.

For group comparison of the network efficiency parameters, non-parametric permutation test with 1000 repetitions was adopted. For each iteration, we reassigned the LGIs of each participant randomly to one of two new groups with the sample size identical as the two groups. For each randomized group, we calculated the global efficiency and local efficiency as well as their normalized integrals (area under the curve divided by the length of integration interval) across a range of sparsity thresholds (10% ≤ *S* ≤ 40%). Differences of the normalized integrals of each efficiency parameter were then calculated from two randomized groups to obtain a null distribution of differences, against which we can compute the P values of the actual difference of normalized integrals of the efficiency parameters between diving experts and controls.

## Results

### Vertex-wise LGI Analysis

**Figure 1:**
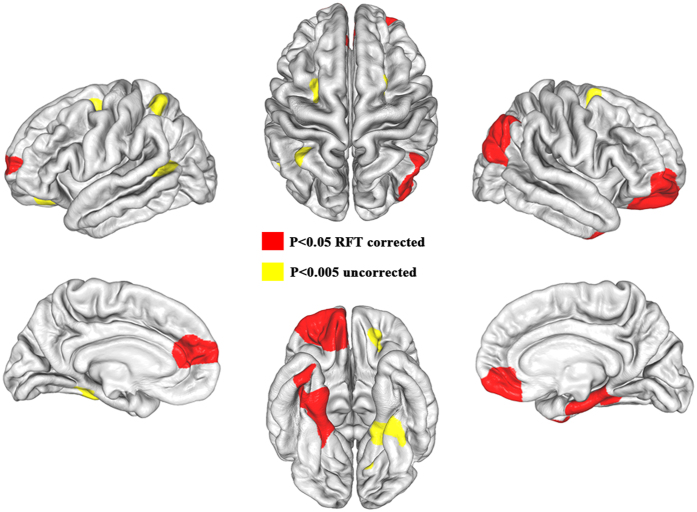
Cortical areas with LGI reductions in diving experts compared with controls. Red clusters are those where significance reached P < 0.05 RFT corrected. Yellow clusters are those where significance reached a looser threshold of P < 0.005 uncorrected.

Compared with controls, we found four clusters where diving experts had significant LGI reductions (P < 0.05 RFT corrected, red clusters in Fig. 1). These clusters involve the bilateral anterior cingulate gyrus, the right superior frontal gyrus and right orbitofrontal cortex, inferior and superior parietal cortex, parahippocampal gyrus, lingual and fusiform gyrus, inferior temporal gyrus and entorhinal cortex. In addition, we found six more clusters showing LGI reductions under a looser threshold of P < 0.005 uncorrected (yellow clusters in Fig. 1), in bilateral caudal middle frontal regions (dorsal premotor cortices), left precentral gyrus, superior parietal cortex, superior temporal sulcus, parahippocampal gyrus, lingual and fusiform gyrus, and orbitofrontal cortex in diving experts. For visualization, regions of difference were projected onto the pial surface of the average template.

**Figure 2:**
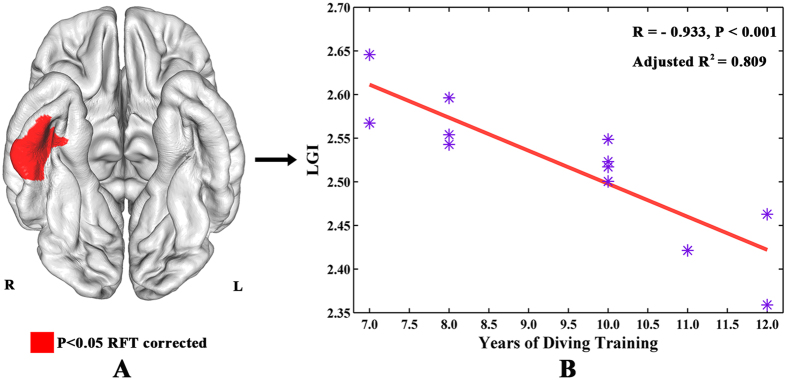
Relationship between LGIs and years of diving training in diving experts. (**A**) Cortical areas showing significant negative correlations between LGIs and years of diving training in diving experts (P < 0.05 RFT corrected). (**B**) Scatter plot showing the negative correlation between years of diving training and the mean LGIs of the significant cluster in vertex-wise correlation analyses.

Within the diving experts, we found a cluster in the medial temporal lobe where LGIs were negatively correlated with years of diving training (P < 0.05 RFT corrected). This cluster involves the right inferior temporal gyrus, fusiform gyrus, parahippocampal gyrus and entorhinal cortex (Fig. 2). Under a looser threshold of P < 0.005 uncorrected, we found one more cluster in the right middle frontal gyrus (premotor cortex) where LGIs were negatively correlated with years of diving training.

### Network Efficiency Analysis

Non-parametric permutation test revealed no significant difference of the normalized integrals of the global efficiency (P = 0.37) and local efficiency (P = 0.48) between diving experts and controls.

## Discussion

Using a surface-based LGI, the present study investigated the cortical gyrification changes in diving experts compared with matched controls. We also performed network efficiency analyses of structural cortical networks inferred from interregional correlation of LGIs to explore whether diving experts are more efficient in information processing. The first major finding was the widespread LGI reduction in diving experts. The cortical areas with LGI reductions were almost symmetrically distributed over both hemispheres, involving the bilateral premotor cortex, bilateral medial temporal lobe, bilateral prefrontal cortex, left precentral gyrus, superior parietal cortex, superior temporal sulcus, and the right inferior parietal cortex. Within the diving experts, vertex-wise correlation analyses revealed two clusters involving the right inferior temporal gyrus, fusiform gyrus, entorhinal cortex and premotor cortex where LGIs were negatively correlated with years of diving training. Next, we documented comparable global and local efficiency in diving experts relative to controls. Taken together, these findings indicate that cortical gyrification changes in diving experts may be the result of long-term diving training which could refine the neural circuitry and might be the anatomical substrate underlying their extraordinary diving performance.

As demonstrated by many previous studies, long-term intensive training could influence brain structure and function along with the underlying connectivity. In the present study, we investigated the specific effect of long-term diving training on cortical gyrification by examining diving experts vs matched controls. Compared with controls, widespread LGI reductions were observed in diving experts. Among the regions showing significant LGI reductions, the differences seen in the posterior parietal cortex, medial temporal lobe and premotor cortex were of particular interest. These regions were shown to be directly or indirectly connected with the vestibular nuclei^29^,^30^, and were considered to be key nodes of the vestibular system, which plays important roles in postural and oculomotor control, bodily perception, spatial cognition^31^, as well as in spatial memory, spatial learning and spatial navigation^32^. The vestibular system codes three dimensional head movements in space by sensing the angular and linear accelerations^31^,^32^. For diving experts, the objective of diving practice is to produce the intended body parabolic trajectory with multiple twists and somersaults, which highlights the importance of postural and oculomotor control as well as a good sense of proprioception and spatial perception^33^. As a closed loop control movements with much complexity, spatial navigation on movements is highly required to achieve the best possible diving. The cluster observed in the medial temporal lobe also involved some visual cortex areas, such as the lingual and fusiform gyrus, which have been found to project to the hippocampal formation and to be very important for visually guided spatial memory and navigation^34^. This finding is in line with the fact that visual spotting is a commonly used technique to assist in spatial orientation and knowing when to come out of the somersaulting dives^33^. In diving experts, the LGIs of this cluster were shown to be negatively correlated with years of diving training, which might reflect the involvement of this region in the storage and retrieval of spatial memories^35^,^36^. On the other hand, the cortical gyrification changes observed in the caudal middle frontal gyrus (dorsal premotor cortex), precentral gyrus, and posterior parietal cortex may be associated with motor functioning given that these regions have the anatomical substrate to influence motor output through direct or indirect connections with the primary motor cortex and the spinal cord^37^,^38^. In fact, the dorsal premotor cortex plays a dominant role in movement selection and planning^39^,^40^ and is considered to be a structure of key importance in motor learning^41^. The posterior parietal cortex, including the inferior parietal lobule and superior parietal lobule, has been involved in intention to perform specific motor acts^42^, preparation and redirection of movements^39^,^40^. The posterior parietal cortex has also been considered to be an important hub where several different types of information are integrated^43^,^44^. The cortical gyrification changes observed in these regions may indicate that diving experts might be more efficient in integrating multi-modal information in this fronto-parietal network to produce an output that reflects the selection, preparation, and execution of movements^45^.

Additional areas of LGI reductions were found in the medial prefrontal cortices (including the anterior cingulate gyrus, medial superior frontal gyrus and medial orbitofrontal gyrus) and lateral orbitofrontal cortex. The medial prefrontal cortex has been shown to play important roles in retrieval of both recent and remote memories^46^ as well as in decision-making including conflict monitoring^47^, error detection^48^ and reward-guided learning^49^. Several lines of evidence indicate that medial prefrontal cortex likely forms and stores schema which maps context and events onto appropriate actions and could direct the correct motor response to a given set of events in light of past experience^50^. Hence, the cortical gyrification changes in the medial prefrontal cortex may relate to the formation of the repertoire of diving experiences through long-term intensive diving training, which enables the diving experts to choose the best diving practice^50^. Additionally, the anterior prefrontal cortex has been involved in the exploration of alternative behavioral options during execution of a prevailing behavioral plan^51^, the cortical gyrification changes in the orbitofrontal cortex may reflect a built-up and maintenance of an optimal diving strategy as a result of exploring and evaluating potential parameters of the timing, strength and coordination during long-term diving practice^51^,^52^,^53^. This interpretation is supported by a previous study reporting that elite divers have higher levels of thrill and adventure seeking, experience seeking and disinhibition than non-divers^54^. However, the exact contribution of cortical gyrification changes observed in the prefrontal cortex to the excellent performance of diving experts remains unclear and needs further investigations.

To test whether diving experts are more efficient in information processing, we performed exploratory network efficiency analyses on the structural networks inferred from interregional correlation of LGI. Unexpectedly, we observed no significant difference in the normalized integrals of global efficiency and local efficiency between diving experts and controls. The negative result of the network efficiency analyses could be due to the small sample size of the current study, which resulted in low statistical power.

Over the years, a number of studies have been conducted to investigate the developmental trajectory of cortical gyrification. Specifically, a longitudinal study on the cortical gyrification development of infants in the first 2 postnatal years reported that the cortical LGI has age-related and marked development with 16.1% increase in the first year and 6.6% increase in the second year^10^. By imaging healthy adolescents twice with a two-year gap, another longitudinal study found that the cortical surface flattens during adolescence^13^. Meanwhile, studies assessing the age effect on the cortical gyrification development in healthy subjects elder than 6 years reported negative correlations between age and LGI^14^,^55^. These studies indicate that the development of the cortical LGI may follow an inverted U-shaped curve^12^ which reaches its peak somewhere between 2 and 6 postnatal years^11^. Such an inverted U-shaped curve of cortical gyrification development is consistent with the two-stage neurodevelopmental process of exuberant proliferation of neurons and their synaptic connections followed by elimination of the excess^15^. The rapid cortical LGI growth of the first two years is likely associated with the increase of dendritic arborization, the growth of the terminal axon arborization, synaptogenesis, and glial proliferation^10^, whereas the subsequent LGI decline may be due to the synaptic pruning as a fine-tuning process of the neural circuitry in preparation for optimal behaviors^1^,^10^. Indeed, age-related changes in synaptic and dendritic arborization may lead to decreasing tensile forces which could result in widening of the sulci and greater curvature of the gyri^13^,^56^. In the current study, both diving experts and controls were all elder than 6 years, and therefore had entered the LGI declining stage. The observed LGI reductions in diving experts indicated that diving experts may either have a faster rate or earlier time of LGI declining or both, which presumably result from increased rate or earlier onset time of synaptic pruning in diving experts. Further exploratory correlation analyses between global LGI and age for each group revealed a significant negative correlation in the control group, whereas such an age-related LGI decline was not found in diving experts (Fig. S1 in the Supplementary Materials). This line of evidence supported the speculation that the LGI reductions in diving experts may result from earlier onset time of synaptic pruning due to intensive diving training. However, this interpretation should be considered as preliminary since the age ranges of the two groups are narrowly distributed. Moreover, we cannot rule out the possibility that increased rate of synaptic pruning is implicated in causing the LGI reductions in the diving experts^57^ given that such cross-sectional correlation analyses cannot reflect the dynamic relationship between LGI and age. Together, the LGI reductions in the diving experts may be related to the fine-tuning process (via synaptic pruning) of neural circuitry.

In addition, the observed cortical gyrification alterations in diving experts can be explained by prevailing theories about the development of cortical gyrification^2^,^4^,^5^. In fact, voxel-based morphometry on the white matter volume of the diving experts revealed significant elevated white matter volume in the thalamus, parahippocampal gyrus, insular cortex and brainstem (Fig. S2 in the Supplementary Materials). The previously reported significant regional inflation in the thalamus and globus pallidus^23^, which are key nodes of the basal ganglia-thalamo-cortical network and have reciprocal connections with the cerebral cortex^58^,^59^, also suggest possible alterations in white matter tract in diving experts. On the other hand, although the present study provided no direct evidence of altered growth rate of different cortical layers, the gray matter density and cortical thickness changes observed in our previous studies are indicative of altered intracortical organization in the diving experts^4^,^21^,^22^. Furthermore, considering the reciprocal thalamocortical connections between the thalamus and different cortical layers^58^,^59^, altered cortical afferent due to changes in the thalamus may result in differential plastic changes in different cortical layers of diving experts^18^,^56^.

This study had some limitations that should be addressed. First, due to the cross-sectional design of the current study, it is indefinite that the cortical gyrification reductions we found were directly caused by diving training. Hence, the major future challenge is to figure out whether the cortical gyrification changes in diving experts were actually induced by diving training, or whether they are inherent prerequisites for the beginning and continuation of diving. Second, the sample size of the current study is relatively small, making the findings of network analysis preliminary in nature. Moreover, the cortical networks were constructed by assessing the interregional structural covariance of cortical gyrification, which only provided an indirect measure of the anatomical networks. Therefore, more comprehensive network analyses with diffusion tensor imaging and also a larger sample size are warranted.

Using a surface-based LGI, we found widespread cortical gyrification reductions in diving experts compared with controls. Within the diving experts, vertex-wise correlation analyses showed significant negative correlations between years of diving training and LGIs of a cluster in the medial temporal lobe. Meanwhile, graph-theoretic network analysis showed comparable global efficiency and local efficiency in diving experts relative to controls. These findings suggests that cortical gyrification reductions in diving experts may be the result of long-term diving training which could refine the neural circuitry (via synaptic pruning) and might be the anatomical substrate underlying their extraordinary diving performance.

## Additional Information

**How to cite this article**: Zhang, Y. *et al*. Effects of Long-term Diving Training on Cortical Gyrification. *Sci. Rep.* **6**, 28243; doi: 10.1038/srep28243 (2016).

## Supplementary Material

Supplementary Information

## Figures and Tables

**Table 1:** Demographic data of the participants.

	Diving experts (n = 12)	Controls (n = 12)
Age (year)^★^	14.58 ± 1.68	14.92 ± 1.38
Education (year)^★^	7.75 ± 1.82	7.92 ± 1.38
Average practice time per day (hour)	6.54 ± 0.38	N/A
Duration of practice (year)	10.12 ± 0.86	N/A
Age of commencement (year)	5.33 ± 0.98	N/A

Note: ^★^Indicates no significant between-group difference using two-sample t-test (p > 0.05).

N/A = not available.
