# Supplementary material for: Effects of Long-term Diving Training on Cortical Gyrification
Source: Sci Rep. 2016 Jun 20;6:28243. doi: 10.1038/srep28243 (PMC4913303; doi:10.1038/srep28243)
Supplement: Supplementary Information [file srep28243-s1.doc]

**Supplementary Materials:**

**Effects of long-term diving training on cortical gyrification**

Yuanchao Zhang, Lu Zhao, Wenwei Bi, Yue Wang, Gaoxia Wei, Alan Evans, Tianzi Jiang

**Figure S1**. Within-group relationship between global LGI and age. We revealed a significant negative correlation between global LGI and age in the controls (P=0.025), whereas such an age-related LGI decline was not found in the diving experts (P>0.3).


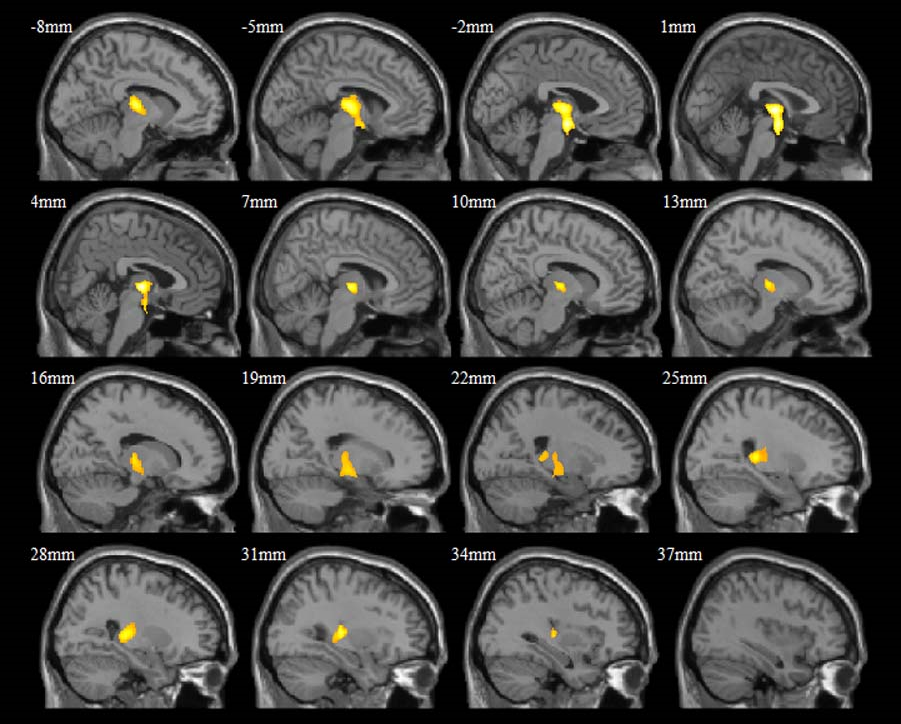


**Figure S2**. Increased white matter volume in diving experts revealed by voxel-based morphometry. Diving experts showed significantly increased white matter volume in thalamus, parahippocampal gyrus, insular cortex and brainstem compared with controls (P<0.05 RFT corrected).
